# Lung adenocarcinoma risk in an area with seasonal high PM_2.5_ exposure: a population-based study in upper northern Thailand

**DOI:** 10.3389/fpubh.2026.1835189

**Published:** 2026-06-16

**Authors:** Pak Thaichana, Patumrat Sripan, Sawaeng Kawichai, Imjai Chitapanarux, Donsuk Pongnikorn, Weerawat Ukranun, Karnchana Daoprasert, Narate Waisri, Puttachart Maneesai, Kittipan Rerkasem, Worawut Srisukkham

**Affiliations:** 1 Office of Research Administration, Chiang Mai University, Chiang Mai, Thailand; 2 Research Institute for Health Sciences, Chiang Mai University, Chiang Mai, Thailand; 3 Chiang Mai Cancer Registry, Faculty of Medicine, Chiang Mai University, Chiang Mai, Thailand; 4 Department of Medical Services, Ministry of Public Health, Bangkok, Thailand; 5 Center Registry Unit, Lampang Cancer Hospital, Lampang, Thailand; 6 Clinical Surgical Research Center, Department of Surgery, Faculty of Medicine, Chiang Mai University, Chiang Mai, Thailand; 7 Department of Computer Science, Faculty of science, Chiang Mai University, Chaing Mai, Thailand

**Keywords:** lung adenocarcinoma, PM_2.5_ exposure, population-based study, Southeast Asia, upper northern Thailand

## Abstract

**Background:**

Air pollution exposure has been associated with increased lung cancer risk globally. However, evidence linking fine particulate matter with an aerodynamic diameter of ≤2.5 micrometers (PM_2.5_) to adenocarcinoma, the most common histological subtype of lung cancer, using the real-world data remains limited in Southeast Asia populations where air pollution levels are rising. This study aimed to investigate the association between average daily PM_2.5_ exposure and lung adenocarcinoma incidence in upper northern Thailand.

**Methods:**

A population-based study was conducted across eight upper northern provinces (103 districts) of Thailand. Daily PM_2.5_ exposure data were linked with lung adenocarcinoma incidence rates obtained from population-based cancer registries during 2013–2017 using patients’ residential areas at the district level. Generalized estimating equations were employed to estimate adjusted incidence rate ratios (IRRs), adjusting for individual-level characteristics (gender and age <65 years) and geographical-level characteristics (urbanization and smoking prevalence).

**Results:**

Among 12,484 lung cancer cases, cell type was identified in 5,465 cases, of which 3,318 (60.7%) were lung adenocarcinoma, with a median age of 64 years (IQR: 57–71). A significant positive association was observed between 10 μg/m^3^ increment in average daily PM_2.5_ exposure and lung adenocarcinoma incidence (adjusted IRR = 1.022, *p* = 0.013). Additional significant associations were found for female (aIRR = 1.126, *p* < 0.001), age <65 years (aIRR = 1.105, *p* < 0.001), and smoking prevalence in the patient’s residing district (aIRR = 0.991, *p* = 0.001).

**Conclusion:**

This finding highlights PM_2.5_ as an important environmental risk factor for lung adenocarcinoma in the region, regardless of smoking prevalence. Our findings support the need for targeted air pollution control policies to reduce incidence of related cancers.

## 1 Introduction

Lung adenocarcinoma, the most common histological subtype of non-small cell lung cancer (NSCLC), represents a distinct biological entity with unique epidemiological characteristics that distinguish it from other lung cancer subtypes (1). Globally, adenocarcinoma accounted for approximately 39% of all lung cancer cases in men and 57% in women in 2020, making it the most prevalent histological subtype worldwide (2).

The knowledge of lung adenocarcinoma in never-smokers has emerged as a critical area of research, given that lung cancer in never-smokers represents the eighth most common cause of cancer death in developed countries (3). Environmental factors, particularly air pollution exposure, have been identified as significant contributors to adenocarcinoma development in this population (4).

Over the past decade (2011–2021), upper northern Thailand has confronted severe air pollution crises due to widespread open burning practices, including agricultural waste burning and forest fires primarily occurring both within Thailand and across the border regions of Myanmar and Laos, which creates transboundary air pollution. These annual haze episodes have emerged as a significant environmental carcinogen contributing to lung cancer development and health risk (5). The toxic haze crisis during the hot dry season, typically from late January to April, has consistently demonstrated ambient concentrations of particulate matter (PM_10_ and PM_2.5_) that dramatically exceed both Thailand air quality standards and WHO guidelines (6, 7). The mountainous topography of the region creates temperature inversion conditions that trap pollutants, leading to prolonged population exposure (8). PM_2.5_ concentrations during the haze period exceeded daily averages of the Pollution Control Department (PCD), Thailand, with urban areas ranging from 24 to 65% above the standard, while rural area concentrations were similarly concerning (9).

Recent research highlights a clear link between fine particulate matter with an aerodynamic diameter of ≤2.5 micrometers (PM_2.5_) exposure and adenocarcinoma, particularly in never-smokers with *Epidermal Growth Factor Receptor* (EGFR)-driven mutations. Functional mouse models have shown the tumor-promoting effects of particulate matter (5), demonstrating how PM_2.5_ exposure activates an inflammatory cascade involving interleukin-6 and others (10). This promotes the growth of lung cells with cancer-causing mutations. The susceptibility of adenocarcinoma to air pollution is rooted in its distinct molecular profile. Environmental carcinogens like polycyclic aromatic hydrocarbons (PAHs) and PM_2.5_ induce DNA damage, oxidative stress, and chronic inflammation (11), creating a favorable environment for malignant transformation in lung tissue (12). These findings underscore the direct carcinogenic role of air pollution based on experimental conditions. The Asia-Pacific region presents a distinctive context for studying PM_2.5_-related lung adenocarcinoma, as it faces some of the world’s highest ambient PM_2.5_ levels driven by industrialization, urbanization, and seasonal agricultural burning (13, 14). While smoking-related lung cancer subtypes have declined in Thailand due to successful reductions in smoking prevalence, the incidence of adenocarcinoma has been rising, particularly among never-smokers and women (15). This trend suggests a potential role for non-smoking risk factors, including environmental exposures (4, 16).

In low-and middle-income countries, particularly in Southeast Asia (SEA), where PM_2.5_ is a significant factor affecting the population’s health, report on effects of PM_2.5_ exposure on lung adenocarcinoma using real-world data remains limited. This study aims to examine the association of PM_2.5_ and lung adenocarcinoma incidence across eight provinces in upper northern Thailand. The findings are expected to provide crucial evidence of this association for shaping effective prevention and control strategies, benefiting both in Thailand and broader society in the SEA region.

## 2 Methods

### 2.1 Study design

This study was conducted across eight provinces in upper northern Thailand (103 districts), which has a permanent population of around 12 million (Supplementary Figure 1). The eight provinces of upper northern Thailand are situated in a flat plain surrounded by high mountain ranges. In this region, biomass burning has been identified as a major source of PM_2.5_ during episodes of haze. This study aimed to assess the associations between PM_2.5_ exposure and the lung adenocarcinoma incidence rate. The data were derived from Lampang cancer hospitals, Lampang province, Thailand. Lung cancer patients who were diagnosed with adenocarcinoma between January 1, 2013, and December 31, 2017, were included in this analysis. Cases with missing information regarding cell type and area of residence were excluded.

### 2.2 Air quality data and exposure assessments

**Figure 1:**
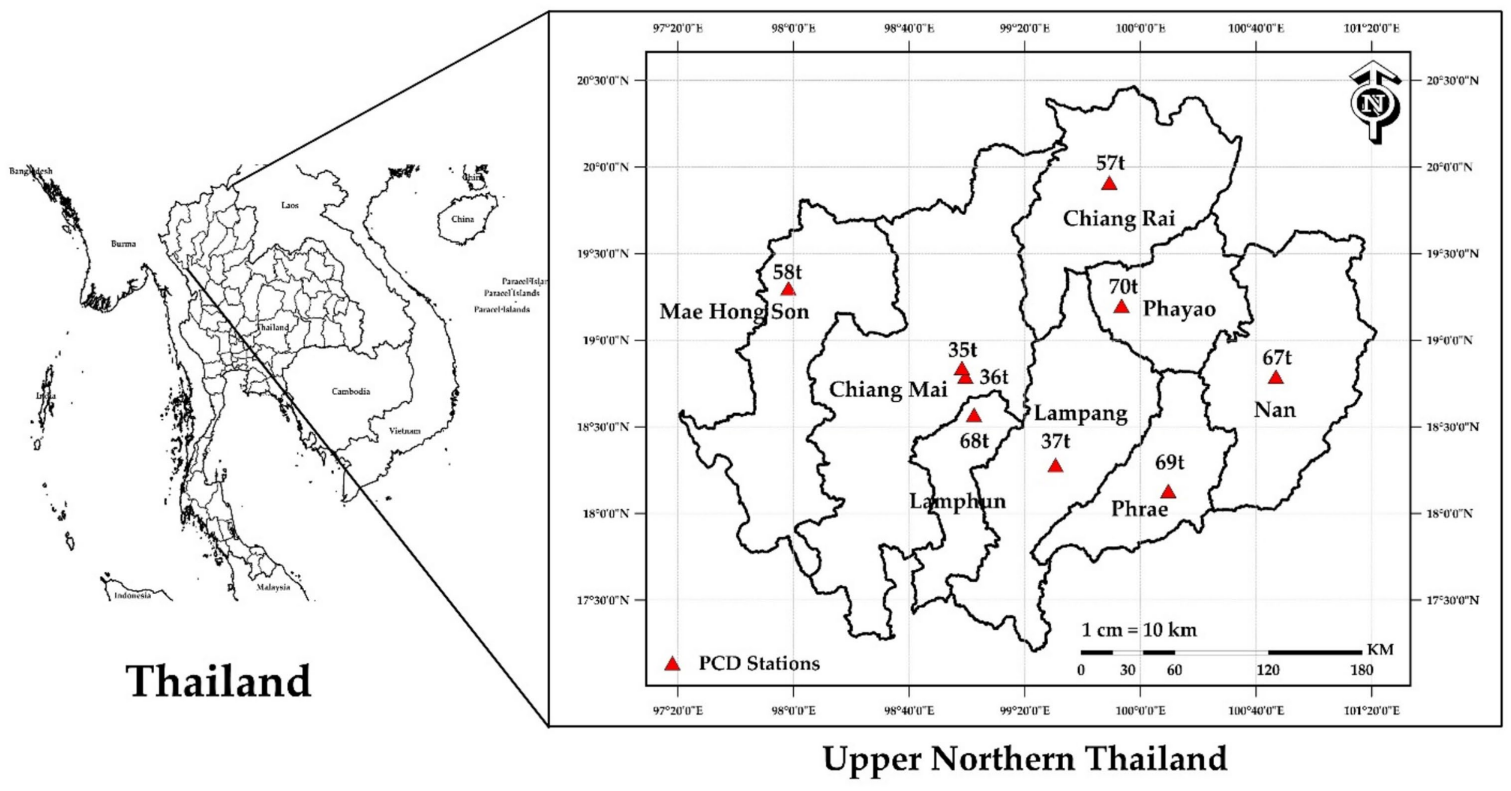
Location of the pollution control Department (PCD)’s monitoring stations in the eight upper northern provinces Thailand.

In this study, air quality data was collected from hourly reports provided by the PCD during the study period. According to the protocol of the national air quality monitoring network, the concentrations of PM_2.5_ across eight provinces in upper northern Thailand (103 districts) were measured at selected national monitoring sites between January 1, 2011, and December 31, 2017. These measurements utilized the Tapered Element Oscillating Microbalance (TEOM) method, with hourly readings aggregated into daily averages. The air pollution data was obtained from air quality monitoring stations located in the Mueang districts of each province (Figure 1). The stations are identified by their codes: 35 t and 36 t in Chiang Mai, 57 t in Chiang Rai, 68 t in Lamphun, 37 t in Lampang, 69 t in Phrae, 67 t in Nan, 70 t in Phayao, and 58 t in Mae Hong Son, respectively. This approach ensured comprehensive and continuous monitoring of PM_2.5_ concentrations in the area. PM_2.5_ concentrations for each district from 2011 to 2017 were calculated using the random forest machine learning method (17). Average daily PM_2.5_ exposure was calculated using 2011–2017 data. Given the low population migration rate in northern Thailand (18), we assumed that the patient exposure to PM_2.5_ with the same average daily before the lung cancer diagnosis. This exposure was studied as a form of chronic exposure toxicity, which shows how air pollution can pose health risks.

### 2.3 Lung cancer data collection

Lung cancer data across eight provinces in upper northern Thailand (103 districts) between 2013 and 2017 were collected from Lampang Cancer Hospital, one of the major government cancer hospitals in the upper northern part of Thailand. Data access was granted from June 2, 2021, to June 2, 2022, following ethics approval from the Research and Human Research Ethics Committee of Lampang Hospital (72/2564). The lung cancer data contains attributes with individual characteristics, including age, gender, and diagnosis data. All personally identifiable information was de-identified prior to data access, and researchers did not have access to patient names, identification numbers, or other direct identifiers. The patient’s permanent address at the district level is linked with the lung cancer data with a municipal map of Thailand. Out of 12,484 lung cancer cases, 7,019 were excluded owing to unidentified cell types. Approximately, half of lung cancer patients (5,465 cases) were analyzed in this study. The group with unidentified cell type was older than the group with known cell type (Supplementary Table 1).

Geographical characteristics, including smoking prevalence and urbanization, were also used in the analysis. The definition of urban and rural areas is based on population density criteria. Districts with a population density of 400 people per square kilometer or more, or those designated as the provincial capital districts, which serve as the economic centers of each province, are classified as urban areas (19, 20). The urban areas included the central district of each province, and additional economic districts included San Kam Phaeng, San Sai, San Pa Tong, and Saraphi in Chiang Mai province and Mae Sai district in Chiang Rai province, Thailand. Smoking prevalence was derived from the Health Data Center (HDC), Ministry of Public Health, Thailand (21).

### 2.4 Statistical analyses

The characteristics of the study population and distribution of daily concentrations of PM_2.5_ data were presented using descriptive statistics. Categorical variables were displayed as frequencies with proportions, while continuous variables were reported as medians with interquartile range (IQR) and the range of maximum and minimum values. The associations between 10 μg/m^3^ increment in average daily PM_2.5_ exposure and lung adenocarcinoma risk were estimated using generalized estimating equations (GEE) with binomial family. A log link function with an exchangeable correlation structure was applied in the model. The incidence rate ratios (IRRs) were estimated for each potential confounder, including individual-level characteristics such as patient age, gender, and geographical-level characteristics such as smoking, and municipality (rural or urban areas). In the multivariable analysis, adjusted incidence rate ratios (aIRRs) with 95% confidence intervals were calculated to estimate the lung adenocarcinoma risk associated with each increase in PM_2.5_ concentration adjusted by significant factors. A backward stepwise selection method was employed to determine the most influential factors affecting the outcomes. Variables with *p*-values greater than 0.2 were excluded from the model in each step, while those with *p*-values less than 0.05 retained. All statistical analyses were conducted using STATA software (StataCorp. 2021. Stata Statistical Software: Release 17. College Station, TX: StataCorp LLC.). All the statistical analyses were calculated using two-sided tests with a 5% level of significance.

### 2.5 Ethics approval

All procedures for this study were reviewed and approved by the Institutional Review Boards of the Faculty of Medicine, Chiang Mai University, Chiang Mai province (SUR-441/2021), and Lampang Cancer Hospital, Lampang province (72/2564), Thailand. The requirement for written informed consent was waived by the ethics committees due to the study’s retrospective design and patient’s data were unidentifiable.

## 3 Results

**Figure 2:**
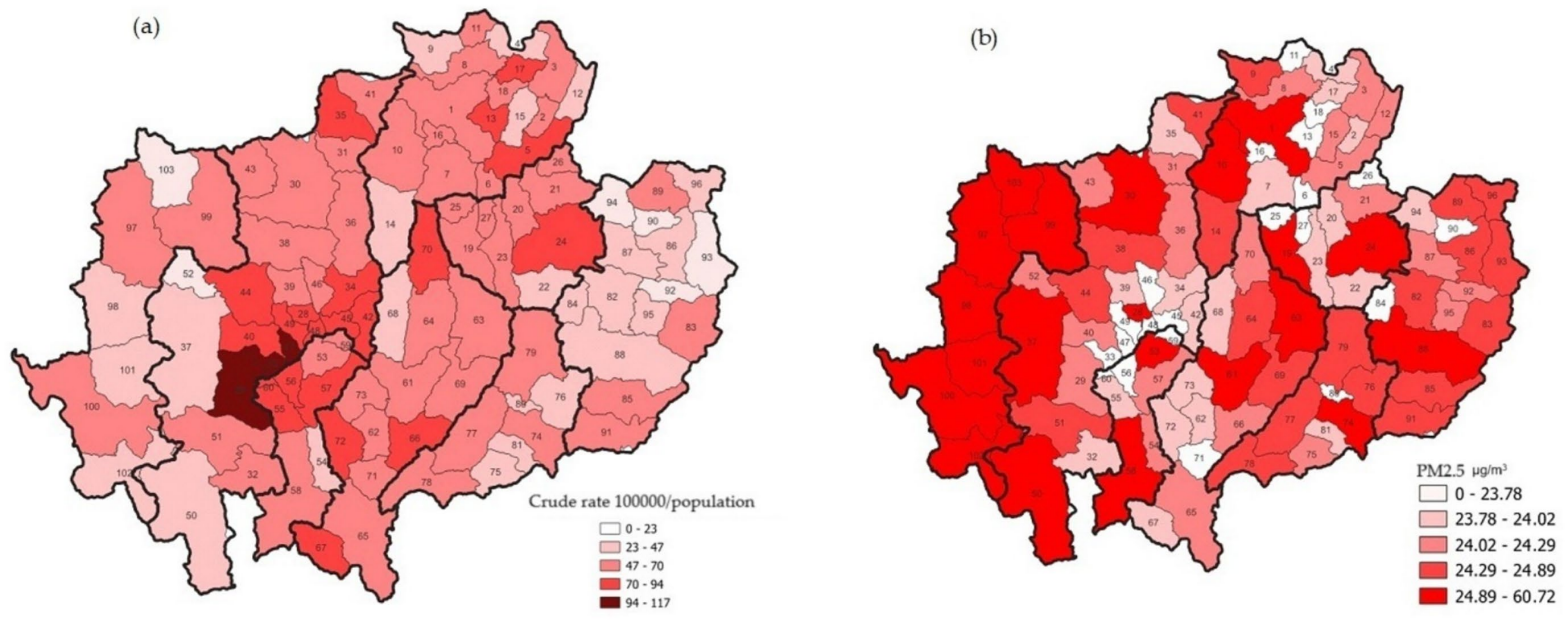
Adenocarcinoma lung cancer incidence **(a)** and average daily PM_2.5_ concentration **(b)** by districts.

Table 1 describes characteristics of lung cancer patients whose the cell type could be identified. Among 5,465 lung cancer, 3,318 (60.7%) identified as lung adenocarcinoma, median age was 64 (IQR: 57–71), and 60.9% diagnosed with metastatic cancer. Incidence of lung adenocarcinoma in upper northern region of Thailand, 2013–2017 and daily PM_2.5_ concentration in upper northern Thailand varied by geographical area (Figure 2); details of the district numbers were described in Supplementary Figure 1.

**Table 1:** Demographic and clinical characteristics of lung cancer patients in whom the cell type could be identified.

Characteristics	*n* (%) or median (IQR)
Sex	
Male	3,272 (59.9)
Female	2,193 (40.1)
Median age (IQR), year	64 (57–71)
Age <65 years	2,886 (52.8)
Age ≥65 years	2,579 (47.2)
Cell Type	
Adenocarcinoma	3,318 (60.7)
Small cell lung cancer	222 (4.1)
Non-small cell lung cancer	428 (7.8)
Squamous cancer	1,008 (18.4)
Large cell cancer	134 (2.4)
Others	355 (6.5)

Continuous data are reported as median (IQR: quartile 1, quartile 3) and categorical data as *n* (%).

PM_2.5_, Particulate matter 2.5 micrometers or less in diameter.

The incidence of lung adenocarcinoma ranged from 0 to 117 per 100,000 population. The three districts with the highest incidence were in Chiang Mai province. The highest crude rate was 117 per 100,000 population in San Pa Tong, followed by 114 per 100,000 population in Doi Lo, and 106 per 100,000 population in Chom Thong. Lung adenocarcinoma was not found in Pang Mapha district in Mae Hong Son province, and only 8 per 100,000 population were found in Song Khwae district in Nan province and Kanlayaniwatthana district in Chiang Mai province. While the daily concentration of PM_2.5_ in northern Thailand ranged from 25.3 to 60.7 μg/m^3^. The highest daily concentration of PM_2.5_ was found in Mueang Chiang Mai district (60.7 μg/m^3^), followed by Mueang Phrae district (31.0 μg/m^3^), and Mueang Phayao district (28.7 μg/m^3^) (Supplementary Table 2).

For the association between lung adenocarcinoma and PM_2.5_ exposure, every 10 μg/m^3^ increase in average daily PM_2.5_ raised the risk of lung adenocarcinoma by 1.022 (95%CI: 1.005–1.040, *p* = 0.013), after adjusting for potential risk factors including female (aIRR = 1.134, *p* < 0.001), age <65 years (aIRR = 1.111, *p* < 0.001), and smoking prevalence (aIRR = 0.990, *p* < 0.001) (Table 2).

**Table 2:** Factor associated with adenocarcinoma lung cancer.

Factors	IRR (95% CI)	*p*-value	aIRR (95% CI)	*p*-value
Individual level				
Female	1.134 (1.087–1.183)	<0.001	1.126 (1.081–1.174)	<0.001
Age <65 years	1.111 (1.064–1.161)	<0.001	1.105 (1.059–1.154)	<0.001
District level				
Urban	1.044 (0.980–1.112)	0.179		
Average daily PM_2.5_ concentration, 10 μg/m^3^ increment	1.027 (1.008–1.047)	0.006	1.022 (1.005–1.040)	0.013
Smoking prevalence	0.990 (0.985–0.996)	<0.001	0.991 (0.986–0.996)	0.001

IRR, Incidence rate ratio; aIRR, Adjusted incidence rate ratio; 95%CI, 95% confidence interval.

## 4 Discussion

This study examined the relationship between PM_2.5_ exposure and lung adenocarcinoma incidence in the upper northern region of Thailand from 2013 to 2017. Our findings reveal a significant association, providing further evidence that links air pollution to lung cancer development.

PM_2.5_ has been classified as a Group 1 carcinogen by the International Agency for Research on Cancer (IARC), with mounting evidence demonstrating its role in promoting adenocarcinoma specifically (4). To the best of our knowledge, our study is the first to report the association between PM_2.5_ exposure and the lung adenocarcinoma incidence in SEA region. Our results, which show an increased risk of adverse health outcomes, are consistent with findings from studies in Taiwan, the United States, and Poland, highlighting the global impact of air pollution (22–24). Although no prior studies in Thailand have specifically examined the effects of PM_2.5_ on the lung adenocarcinoma incidence, a study of the upper northern population found that air pollution factors can affect the risk patterns of lung cancer mortality in upper northern Thailand (25). Moreover, exposure to an annual mean PM_2.5_ concentration over 25 μg/m^3^ was associated with an increased mortality rate (26, 27), which indirectly supports our finding regarding the adverse effects of air pollution on the incidence rate. Furthermore, in addition to its carcinogenic effects, PM_2.5_ exposure has been consistently associated with increased all-cause mortality, highlighting its broader public health impact beyond cancer risk. A large-scale health impact assessment across European cities estimated substantial premature mortality attributable to long-term air pollution exposure, reinforcing the global burden of PM_2.5_-related health outcomes (28).

The finding from our analysis was the significant positive association between average daily PM_2.5_ exposure and lung adenocarcinoma incidence (IRR = 1.022, 95% CI: 1.005–1.040, *p* = 0.013). While the magnitude appears small, this finding is epidemiologically meaningful when considering population-level impacts. Our results align with recent meta-analytical evidence demonstrating that increase in PM_2.5_ concentrations is associated with a statistically significant 9% increase in risk for lung cancer incidence or mortality (29). However, district-level PM_2.5_ estimates derived from fixed monitoring stations and random forest modeling represent an ecological-level exposure proxy that cannot fully capture within-district heterogeneity. Moreover, it should be noted that the GEE model accounted for correlations and clustering within districts, yielding statistically robust marginal population averages for the districts where patients resided, but individual-level exposure could not be assessed. In addition, although we were able to infer potential factors at individual level, the results could still be biased by unmeasured confounders. Nevertheless, given the limited long-term PM_2.5_ data available, our study is the first in SEA to evaluate the long-term effects of PM_2.5_ on health outcomes such as lung cancer incidence (30).

The biological plausibility of this association is supported by emerging mechanistic research showing that PM_2.5_ levels were significantly associated with the incidence of EGFR-driven lung cancer cases (5), which are particularly relevant to adenocarcinoma pathogenesis. PM_2.5_ exposure is linked to increased lung cancer incidence, often driven by EGFR and *Kirsten rat sarcoma viral oncogene* mutations. These mutations may impact on important regulatory genes, such as TP53, a tumor suppressor gene essential for cell cycle control. Furthermore, PM_2.5_ exposure induces multiple cell death pathways, including apoptosis, autophagy, and ferroptosis (31, 32). EGFR mutation status was unavailable in routine cancer registry data. Consequently, the independent contributions of PM_2.5_ exposure and EGFR mutation status cannot be fully disentangled from the current data. However, the higher adenocarcinoma risk observed among females and age <65 years may partly reflect the underlying distribution of EGFR-mutant subtypes rather than direct PM_2.5_ effects, as EGFR-mutant adenocarcinoma disproportionately affects never-smokers, females, and age <65 years individuals—a demographic profile consistent with our findings (5, 33). Future studies should employ individual-level cohort designs with longer follow-up periods. Integration of molecular tumor profiling including EGFR into cancer surveillance systems would enable disentanglement of PM_2.5_-attributable risk by molecular subtype and clarify whether PM_2.5_ preferentially promotes EGFR-mutant adenocarcinoma or acts through mutation-independent pathways. Incorporating personal exposure monitoring, genetic susceptibility markers, and lifestyle factors, alongside multi-center prospective approaches, would further strengthen generalizability and inform precision-prevention strategies.

In our study, the higher adenocarcinoma incidence rates observed in females (IRR = 1.126) align with global epidemiological patterns, where lung adenocarcinoma consistently shows higher incidence rates in females, particularly among never-smokers (15). Our study also indicated higher adenocarcinoma incidence rates in areas with lower smoking prevalence (IRR = 0.991). Although adenocarcinoma has been reported as the most common lung cancer cell type in never-smokers (15), individual-level smoking status was unavailable for our study. Therefore, we could not replicate the evidence from a study in Taiwan, which demonstrated that lung adenocarcinoma incidence was significantly higher among females than among males regardless of age, tumor stage, and smoking status (34).

Our findings are particularly relevant to the broader SEA context, where seasonal biomass burning contributes to persistently elevated PM_2.5_ levels (35, 36). In our study, the long-term effects of PM_2.5_ was under the assumption that patients were exposed to the same average daily PM_2.5_ level from birth until the diagnosis of lung cancer, given the low population migration rate in northern Thailand (18). Although the association between PM_2.5_ exposure and lung adenocarcinoma may be applicable to populations in other SEA countries with similar seasonal burning patterns, including Vietnam, Cambodia, Laos and Malaysia (37, 38) in which long-term PM_2.5_ data were limited (30), this assumption should be interpreted with caution and explicitly addressed.

There are some limitations that should be acknowledged in interpreting these results. First, the PM_2.5_ exposure assessment, while covering period may not capture the full latency period relevant to lung cancer development, which typically spans decades. A study in China reported that lung cancer incidence was significantly correlated with PM_2.5_ concentrations at various yearly time lags (from 3 to 9 years, except for lag 6), with the strongest correlation observed at lag 9 (39). The oldest PM_2.5_ data used in our study were from 2011 and were estimated using machine learning for some districts where data were limited. Therefore, we evaluated the long-term effects of PM_2.5_ using the average daily PM_2.5_ level, based on the specific assumption of a low population migration rate, which may not apply to some areas. Second, the findings of this study could only be analyzed and interpreted for approximately half of the lung cancer patients, for whom cell type could be identified. This represents a limitation owing to unavailable pathological results, particularly among patients diagnosed at a late stage of lung cancer, which is common in low-and middle-income countries. Furthermore, potential confounding factors such as occupational exposures, indoor air pollution, radon, genetic susceptibility, and the detailed amount of daily smoking (expressed as pack-years), and other ambient air pollutants including NO_2_ were not available for analysis. Lastly, the most recent data were available up to 2017 because the time delay in population-based cancer registries is usually around 2–3 years. Moreover, the study was conducted during the SARS-CoV-2 outbreak, which led to further delays as impact to cancer registries worldwide (40). However, data collected before the pandemic avoided the confounding influence of the pandemic on our findings.

These findings have important implications for lung cancer prevention and air quality management in upper northern Thailand. The association between PM_2.5_ exposure and lung adenocarcinoma incidence, combined with the known high lung cancer burden in this region, underscores the urgent need for comprehensive air quality improvement strategies. Given that PM_2.5_ exposure may particularly affect lung cancer in people who never smoked, particularly among women, with significant differences in diagnosis stages among EGFR patients (31), screening strategies should consider environmental exposure history alongside traditional risk factors.

Beyond lung cancer, PM_2.5_ exposure poses significant health risks across vulnerable populations. In older adults, long-term PM_2.5_ exposure has been associated with increased cardiovascular risk, respiratory diseases, and cognitive decline (41, 42). Children are particularly susceptible to PM_2.5_-related health effects, including reduced lung function development, and increased asthma prevalence (43, 44). Pregnant women exposed to elevated PM_2.5_ levels face increased risks of adverse birth outcomes, including preterm birth, low birth weight, and small for gestational age infants (45, 46). These associations highlight the multi-generational impact of air pollution and emphasize the critical importance of implementing targeted air quality interventions to protect vulnerable populations throughout upper northern Thailand, particularly during seasonal haze episodes when PM_2.5_ concentrations reach hazardous levels.

In conclusion, the finding of this study provides evidence for an association between PM_2.5_ exposure and lung adenocarcinoma incidence in upper northern Thailand, a region experiencing seasonally high PM_2.5_ levels. Although individual factors such as a slight increase in risk in younger age groups and females were observed, the overall incidence of lung adenocarcinoma appears to be driven by environmental factors like PM_2.5_ exposure. Using the real-world data, this research confirms PM_2.5_ contributes to lung adenocarcinoma regardless of smoking prevalence. Therefore, regional authorities should prioritize comprehensive air quality management to reduce the incidence of related cancers.

## Data Availability

The raw data supporting the conclusions of this article will be made available from the authors upon reasonable request.
